# Exploration of the Molecular Mechanism for Lipoprotein Lipase Expression Variations in SH-SY5Y Cells Exposed to Different Doses of Amyloid-Beta Protein

**DOI:** 10.3389/fnagi.2020.00132

**Published:** 2020-05-12

**Authors:** Jingzhu Zhang, Yufan Liu, Sihui Wang, Ran Que, Weidong Zhao, Li An

**Affiliations:** ^1^Department of Nutrition and Food Hygiene, School of Public Health, China Medical University, Shenyang, China; ^2^China Medical University-The Queen’s University of Belfast Joint College, China Medical University, Shenyang, China; ^3^Department of Developmental Cell Biology, Key Laboratory of Cell Biology, Ministry of Public Health, China Medical University, Shenyang, China; ^4^Key Laboratory of Medical Cell Biology, Ministry of Education, China Medical University, Shenyang, China

**Keywords:** Alzheimer’s disease, amyloid-β, lipoprotein lipase, histone deacetylase 2, histone deacetylase 3, microRNA-29a, peroxisome proliferator-activated receptor γ

## Abstract

Progressive accumulation of amyloid-β (Aβ) plaques in the brain is a characteristic pathological change in Alzheimer’s disease (AD). We previously found the expression of lipoprotein lipase (LPL) was increased in SH-SY5Y cells exposed to low-dose Aβ and decreased in cells with high-dose Aβ exposure, but the molecular mechanism is still unclear. Based on previous studies, the opposite regulation of histone deacetylase2 (HDAC2) and HDAC3 on LPL expression probably explain the above molecular mechanism, in which microRNA-29a and peroxisome proliferator-activated receptor γ (PPARγ) may be involved. This study further revealed the mechanism of HDAC2 and HDAC3 on conversely regulating LPL expression. The results showed that HDAC2 down-regulated microRNA-29a by decreasing histone acetylation (Ace-H3K9) level in its promoter region, subsequently increasing LPL expression directly or through PPARγ/LPL pathway; HDAC3 decreased LPL expression through inhibiting Ace-H3K9 levels in LPL and PPARγ promoter regions and up-regulating microRNA-29a. This study also found that with increasing concentrations of Aβ in cells, HDAC2 and HDAC3 expression were gradually increased, and Ace-H3K9 levels in LPL and PPARγ promoter region regulated by HDAC3 were decreased correspondingly, while Ace-H3K9 levels in microRNA-29a promoter region modulated by HDAC2 were not decreased gradually but presented a U-shaped trend. These may lead to the results that a U-shaped alteration in microRNA-29a expression, subsequently leading to an inverse U-shaped alteration in PPARγ or LPL expression. In conclusion, HDAC2 and HDAC3 at least partly mediate LPL expression variations in different concentrations of Aβ exposed SH-SY5Y cells, in which microRNA-29a and PPARγ are involved, and the histone acetylation level in microRNA-29a promoter region plays a key role.

## Introduction

Alzheimer’s disease (AD) is a progressive irreversible neurodegenerative disorder characterized by cognitive dysfunction and behavioral impairment. It is known that the molecular pathogenesis of AD is complex, involving the interplay of multiple factors (Lane et al., 2018). However, there is no doubt that amyloid-β (Aβ) plaques’ abnormal deposition in the brain is a characteristic pathological change in AD (Castellani et al., 2019). Accordingly, researching Aβ is quite necessary to explore the underlying mechanism of AD pathogenesis.

Lipoprotein lipase (LPL), a member of the lipase gene family, is widely distributed in adipose, heart, and skeletal muscle tissue, as well as in the brain (Cruciani-Guglielmacci and Magnan, 2017). Undergoing a series of transcription, translation, and post-translational processing events, LPL is transported to the different compartments of the Golgi apparatus (Mead et al., 2002). After sorting in the trans-Golgi apparatus, LPL is first delivered to the secretory vesicles, subsequently to cell surface primarily and to the lysosomes slightly (Mead et al., 2002). The genetic analysis has provided a correlation between the common mutations in the LPL gene and AD (Ren and Ren, 2016; Tan et al., 2017). Moreover, LPL has been found to aggregate near senile plaques in the brain of AD (Nishitsuji et al., 2011; Ma et al., 2013). However, the alteration of the LPL expression level in the AD brain remains controversial (Baum et al., 2000; Wang and Eckel, 2012; Gong et al., 2013). Some studies showed that LPL expression was increased in the hippocampus and cerebral cortex of AD patients (Blain et al., 2006; Wang and Eckel, 2012). While Gong et al. (2013) found that the expression of LPL was distinctly decreased in the hippocampal dentate gyrus of AD patients. Our previous study indicated that the expression of LPL was significantly increased in SH-SY5Y cells exposed to a low concentration of Aβ (2 μM), but obviously decreased in cells with a high concentration of Aβ (10 μM) exposure (Zhang et al., 2018). It is speculated that LPL expression increases first and then decreases with the ever-increasing accumulation of Aβ in AD brain. Nevertheless, the molecular mechanism for the regulation of LPL expression by Aβ is still inconclusive.

Histone deacetylases (HDACs), a key enzyme for modification of histone acetylation, can lead to compaction of chromatin and gene repression by inhibiting the acetylation level of histone in the gene promoter region (Gupta et al., 2020). Evidence has indicated that HDACs, especially HDAC2 and HDAC3, are probably implicated in the regulation of Aβ on LPL expression (Chen et al., 2007; Lee et al., 2007; Fischer, 2014). In our previous study, HDAC2/3 expression was significantly increased in SH-SY5Y cells exposed to both low-dose (2 μM) and high-dose (10 μM) Aβ (Zhang et al., 2018). We also found that the expression of LPL was down-regulated in HDAC2-silenced SH-SY5Y cells but up-regulated in HDAC3-silenced cells (Zhang et al., 2018). It is suggested that HDAC2/3 may play a crucial role in the change of LPL expression caused by different concentrations of Aβ, and the regulatory mechanisms of HDAC2 and HDAC3 on LPL expression may be different, however, this needs to be confirmed. Additionally, it remains to be further studied whether HDAC2 or HDAC3 affects the level of histone acetylation in the promoter region of LPL or not.

MicroRNA-29a (miR-29a) can regulate the post-transcription of LPL through base pairing with the 3′ untranslated regions (UTRs) of LPL mRNA (Chen et al., 2011). Our previous studies showed that the expression of miR-29a was decreased in SH-SY5Y cells exposed to low-dose Aβ (2 μM) and increased in cells with high-dose Aβ (10 μM) exposure, corresponding to the change of LPL expression respectively induced by 2 μM and 10 μM Aβ (Zhang et al., 2018). Meanwhile, miR-29a expression can be down-regulated by HDAC2 and up-regulated by HDAC3 in SH-SY5Y cells (Zhang et al., 2018). Therefore, miR-29a may have a pivotal effect on HDAC2- or 3-mediated alteration of LPL expression induced by Aβ. Moreover, the opposite regulation of HDAC2 and 3 on miR-29a expression implies that the regulatory mechanisms of HDAC2 and HDAC3 on miR-29a are different and HDAC2 is likely to inhibit the transcriptional activity of miR-29a by reducing the level of histone acetylation in the promoter region.

The nuclear receptor peroxisome proliferator-activated receptor γ (PPARγ), a ligand-activated transcription factor, forms heterodimers with retinoid X receptors and recognizes specific DNA sequences called PPAR response elements in LPL gene, thereby up-regulating the expression of LPL (Goto, 2019; Kotha et al., 2020). Silencing HDAC2 causes a down-regulation of PPARγ expression in Bone marrow mesenchymal stem cells (Lee et al., 2014), whereas inhibiting HDAC3 results in an up-regulation PPARγ expression in mouse embryonic fibroblast cells or Human embryonic kidney cells (Jiang et al., 2014). However, it is still unclear whether PPARγ is involved in HDAC2/3-mediated alteration of LPL expression induced by Aβ in neurocytes or not.

In the present study, we have proposed and verified a working hypothesis that the regulatory mechanism, the increased first and then decreased of LPL expression with increasing Aβ concentration in SH-SY5Y cells, is related to the opposite regulation of HDAC2 and HDAC3 on LPL expression, in which miR-29a and PPARγ may be involved, especially miR-29a probably plays a key role. This study will provide a new insight into the role of Aβ in the possible mechanisms for LPL regulation.

## Materials and Methods

### Reagents

Aβ_25–35_, a toxic fragment of the full-length Aβ peptide, was purchased from American Peptide Company (131602-53-4, United States), which needs to be solubilized in sterile water and aggregated at 37°C for 7 days before use. The siRNA transfection reagent was purchased from Guangzhou RiboBio Company Limited (C10511-1, Guangzhou, China). The Simple ChIP Enzymatic Chromatin IP Kit was obtained from Cell Signaling Technology (9003S, BOS, USA), and rabbit anti-acetylation of histone 3 lysine 9 (Ace-H3K9) polyclonal antibody for ChIP was from Merck Millipore (06942, Darmstadt, Germany). The rabbit anti-PPARγ polyclonal antibody (D262458), rabbit anti-HDAC2 polyclonal antibody (D155199), and rabbit anti-HDAC3 polyclonal antibody (D260481) were purchased from Bio Basic Inc. (Canada); rabbit anti-LPL polyclonal antibody (sc-32885) and rabbit anti-β-actin polyclonal antibody (sc-130656) were obtained from Santa Cruz Biotechnology (Santa Cruz, CA, USA); Goat anti-rabbit immunoglobulin G (lgG) secondary antibody was from Shanghai Sangon Biotech Company Limited (D111018, Shanghai, China). Cell culture medium was purchased from American Hyclone Inc. (SH30023.01B, USA).

### Cell Culture

As previously reported (Zhang et al., 2017), human neuroblastoma cells (SH-SY5Y, Chinese academy of sciences cell bank, KCB2006107YJ, Kunming, China) were cultured at 37°C; with 5% CO_2_ in DMEM/F12 (1:1) media with 10% fetal bovine serum (SV30087.02, American Hyclone Inc.), and 1% penicillin-streptomycin (15140122, GIBCO-BRL). Cells were used in the following experiments, and each experiment was conducted in duplicate and repeated three times. All experiments were approved by China Medical University, which complies with international biosecurity standards.

### Small Interfering RNA (siRNA)

HDAC2 and HDAC3 siRNA duplex (Guangzhou RiboBio Company Limited) were respectively used to interfere with endogenous HDAC2 and HDAC3 mRNA expression. Transfection of siRNA was carried out as we have described in detail previously (Zhang et al., 2017). The following siRNA oligos were used: HDAC2: 5′-TCCGTAATGTTGCTCGATG-3′; HDAC3: 5′-GCATTGATGACCAGAGTTA-3′. The non-specific siRNA (scrambled siRNA; Guangzhou RiboBio Company Limited) was used as control. The expression of PPARγ mRNA and protein were measured by quantitative reverse transcriptase-polymerase chain reaction (qRT-PCR) and Western blot analyses, respectively. The levels of Ace-H3K9 in the promoter region of LPL, PPARγ, and miR-29a were investigated by Chromatin immunoprecipitation-polymerase chain reaction (ChIP-PCR) assay.

### qRT-PCR Analyses

Cells were seeded in six-well culture microplates at a density of 1 × 10^5^ cells/well in 2 ml of antibiotic-free normal growth medium and incubated for 24 h with Aβ_25–35_ at a final concentration of 0 (blank control), 2.5, 5, 7.5 or 10 μM. Subsequently, the cells were collected and used for HDAC2, HDAC3, LPL, PPARγ mRNA, and miR-29a expression analyses by qRT-PCR. Total mRNA and microRNAs extraction, reverse transcription, as well as Real-Time PCR reactions, were performed as we have described previously (Zhang et al., 2017). The primer sequences for Real-Time PCR are listed as follows: homo LPL, forward: 5′-CCGCCGACCAAAGAAGAGAT-3′, reverse: 5′-TAGCCACGGACTCTGCTACT-3′ (117 bp product); homo HDAC2 forward: 5′-AGGTTGAAGCCATTCTCCTG-3′, reverse: 5′-ATCCCAGCACTTTGGAAGG-3′ (179 bp product); homo PPARγ, forward: 5′-TCTCTCCGTAATGGAAGACC-3′, reverse: 5′-GCATTATGAGACATCCCCA-3′ (474 bp product); homo HDAC3 forward: 5′-GAGGGATGAACGGGTAGACA-3′, reverse: 5′-CAGGTGTTAGGGAGCCAGAG-3′ (137 bp product); β-actin, forward: 5′-CATCCGTAAAGACCTCTATGCCAAC-3′, reverse: 5′-ATGGAGCCACCGATCCACA-3′ (171 bp product); hsa-miR-29a-3p, forward: 5′-CTAGCACCATCTGAAATCGGTTA-3′, reverse: 5′-CGCTTCACGAATTTGCGTGTCAT-3′; U6, forward: 5′-GCTTCGGCAGCACATATACTAAAAT-3′, reverse: 5′-CGCTTCACGAATTTGCGTGTCAT-3′. β-actin and U6 were respectively used as internal controls to normalize mRNA and microRNA levels. Data were analyzed by the comparative C_T_ method (also known as the 2^−ΔΔCT^ method).

### Western Blot Analyses

Cell treatment procedures were performed as described for the qRT-PCR analyses. The same method was used as the one employed in our previous study (Zhang et al., 2017) to collect samples and then to perform Western blot. Briefly, RIPA buffer containing 0.1% protease inhibitor (Amerso, USA) was used to homogenize collected cell samples. The protein concentrations in the supernatants were measured by using BCA Protein Assay Kits (CW0014, CwBio, Inc., Beijing, China). The following antibodies were used: rabbit anti-HDAC2 (1:1,000), anti-HDAC3 (1:1,000), anti-LPL (1:1,000), anti-PPARγ (1:1,000) or anti-β-actin (1:1,000) antibody. β-actin was used as a reference standard to normalize protein levels. The results for Western blot were expressed as folds of control.

### Transfection of microRNA Mimic and Inhibitor

Human miR-29a mimic and inhibitor (Guangzhou RiboBio Company Limited China) were used to up-regulate and down-regulate the expression of miR-29a in cells, respectively. miR-29a mimic sequence: 5′-UAGCACCAUCUGAAAUCGGUUA-3′, anti-sequence: 5′-AUCGUGGUAGACUUUAGCCAAU-3′; and miR-29a inhibitor sequence: 5′-mUmAmAmCmCmGmAmUmUmUmCmAmGmAmUmGmGmUmGmCmUmA-3′ (mN, 2′-O-methyl ribose). Transfection of miR-29a mimic or inhibitor in cells was performed as we have described in detail previously by using ribo FECT™ CP Transfection Kit (Guangzhou RiboBio Company Limited; Zhang et al., 2018). The micrOFFR miRNA mimic control and micrOFFR miRNA inhibitor control (Guangzhou RiboBio Company Limited) were used as controls. The transfected cells were collected to measure PPARγ mRNA and protein expression levels by the above methods.

### ChIP-PCR Assay

ChIP-PCR was performed according to the manufacturer’s protocol provided in the Enzymatic Chromatin IP Kit (9003, Cell Signaling Technology, Danvers, MA, USA) as previously described in detail (Xing et al., 2019). Immunoprecipitation was performed using anti-Ace-H3K9 antibodies. Immunoprecipitated DNA was analyzed by PCR using the following primers: homo LPL, forward: 5′-GG GCCCCCGGGTAGAGTGG-3′, reverse: 5′-CACGCCAAGGCT GCTTATGTGACT-3′; homo PPARγ, forward: 5′-CTACTG TACAGTTCACGC-3′, reverse: 5′-GGGAGAGGTGGGAATA AA-3′; homo miR-29a, forward: 5′-ACGACAGATTGAAGGC CTGGG-3′, reverse: 5′-GGTGCTCTTCCCCAATCA-3′. Serial dilutions of input DNA were used as Input Samples for each primer pair. The equation shown below was used to calculate the IP efficiency. Percent Input = 2%*2^(C[T]2% Input Sample− C[T] IP Sample)^, C[T] = C_T_ = Threshold cycle of PCR reaction. The results for ChIP-PCR were expressed as folds of control.

### Statistical Analyses

Mean ± standard deviation (SD) was used to present graphically all data. Statistical analysis of the data between the two groups was performed with the Student’s *t*-test. Individual comparisons among more groups were performed by one-way analyses of variance including appropriate variables followed by Fisher’s least significant difference multiple comparison *post hoc* tests in SPSS 20.0 software for Windows (Chicago, IL, USA). Results were considered statistically significant when probability values less than 0.05.

## Results

### Ace-H3K9 Levels in the Promoter Region of LPL, miR-29a and PPARγ in HDAC2- or HDAC3-Silenced SH-SY5Y Cells

**Figure 1:**
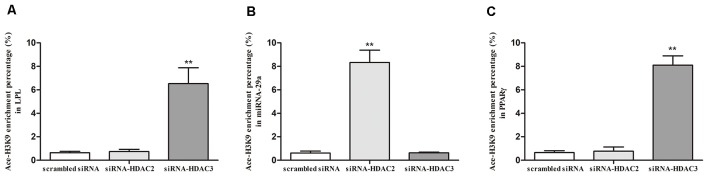
The effects of silencing HDAC2 or HDAC3 on Ace-H3K9 levels in the promoter region of LPL, miR-29a and PPARγ in SH-SY5Y cells. ChIP-PCR assay was used to measure the levels of Ace-H3K9 in the promoter region of LPL **(A)**, miR-29a **(B)**, and PPARγ **(C)** in cells (*n* = 6; mean ± SD; Student’s *t*-test; **p < 0.01 vs. scrambled siRNA group).

To explore the regulatory mechanism of LPL, miR-29a, and PPARγ expression by HDAC2 and HDAC3, we investigated the Ace-H3K9 levels in the promoter region of LPL (Figure 1A), miR-29a (Figure 1B) and PPARγ (Figure 1C) in HDAC2- or HDAC3-silenced cells. Compared with control, the level of Ace-H3K9 in the promoter region of miR-29a was significantly increased (*p* < 0.01), but Ace-H3K9 levels in the promoter region of LPL and PPARγ were unaltered in HDAC2-silenced cells (*p* > 0.05). However, in HDAC3-silenced cells, Ace-H3K9 levels in the promoter region of LPL and PPARγ were significantly increased (*p* < 0.01), but the level of Ace-H3K9 in the promoter region of miR-29a was unchanged (*p* > 0.05) compared with control.

### Alterations of PPARγ Expression in SH-SY5Y Cells With Different Treatments

#### PPARγ Expression in Aβ-Exposed Cells

**Figure 2:**
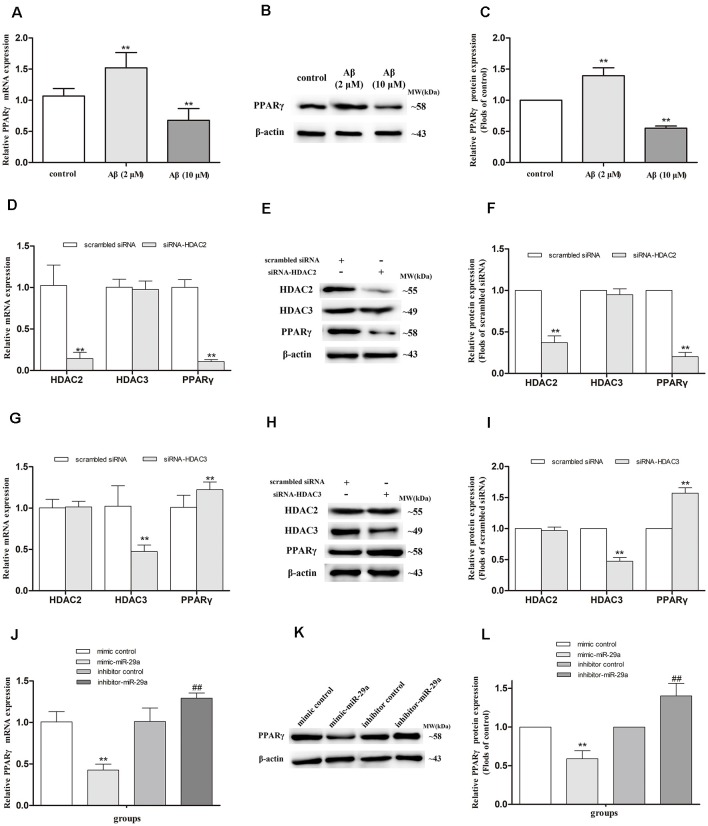
Alterations of PPARγ expression in SH-SY5Y cells with different treatments. The relative expression of PPARγ mRNA and protein were respectively analyzed by qRT-PCR and Western blot in cells exposed to 2 or 10 μM amyloid-β (Aβ; **A–C**), with HDAC2/3-silencing treatment **(D–I)**, and treated with miR-29a mimic or inhibitor [**J,K**; *n* = 6; mean ± SD; One-way ANOVA followed by LSD multiple comparison tests, Student’s *t*-test; **p < 0.01 vs. control **(A,C)**, scrambled siRNA **(D,F,G,I)** or mimic control **(J,L)**, ^##^*p* < 0.01 vs. inhibitor control].

To investigate whether PPARγ mediates the regulation of Aβ on LPL expression in SH-SY5Y cells, we detected the expression of PPARγ mRNA (Figure 2A) and protein (Figures 2B,C) in cells separately exposed to 2 and 10 μM Aβ. Compared with control, the expression of PPARγ mRNA and protein were elevated (*p* < 0.01) in cells exposed to 2 μM Aβ, but significantly reduced (*p* < 0.01) in cells with 10 μM Aβ exposure.

#### PPARγ Expression in HDAC2- or HDAC3-Silenced SH-SY5Y Cells

HDAC2 and HDAC3 siRNA duplex were used to further determine whether HDAC2/3 regulates PPARγ expression in SH-SY5Y cells. Compared with control (scrambled siRNA), the expression of PPARγ mRNA and protein were decreased in HDAC2-silenced cells (*p* < 0.01), but significantly increased in HDAC3-silenced cells (*p* < 0.01; Figures 2D–I).

#### PPARγ Expression in SH-SY5Y Cells Treated With miR-29a Mimic or Inhibitor

To further investigate whether miR-29a regulates the expression of PPARγ, miR-29a expression was respectively interfered with by miR-29a mimic and inhibitor in SH-SY5Y cells (Figures 2J–L). Compared with control (mimic or inhibitor control), the expression of PPARγ mRNA and protein were distinctly reduced in cells with miR-29a mimic treatment (*p* < 0.01) and markedly elevated in cells with miR-29a inhibitor treatment (*p* < 0.01).

### Dose-Effect Relationship Between Aβ Concentration and the Expression Levels of HDAC2/3 in SH-SY5Y Cells

**Figure 3:**
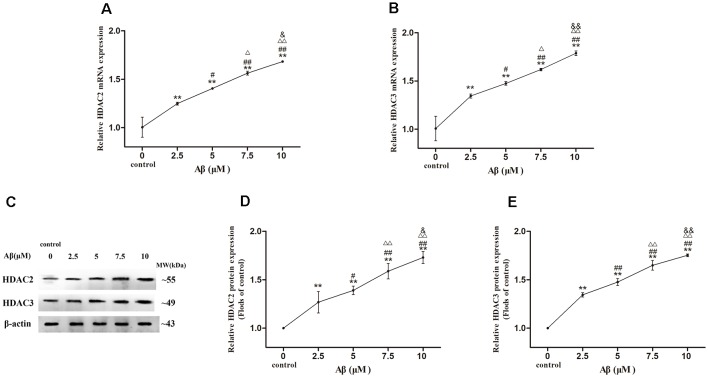
Dose-effect relationships between Aβ concentration and HDAC2/3 expression levels in SH-SY5Y cells. The relative expression of HDAC2 and HDAC3 mRNA **(A,B)** and protein **(C–E)** were analyzed by qRT-PCR and Western blot, respectively. β-actin was used as a reference standard [*n* = 6; mean ± SD; One-way ANOVA followed by LSD multiple comparison tests; **p < 0.01 vs. control (0 μm Aβ); ^#^*p* < 0.05, ^##^*p* < 0.01 vs. 2.5 μm Aβ; ^Δ^*p* < 0.05, ^ΔΔ^*p* < 0.01 vs. 5 μm Aβ; ^&^*p* < 0.05, ^&&^*p* < 0.01 vs. 7.5 μm Aβ].

More exposure concentrations of Aβ were applied for exploring the regulatory mechanism of HDAC2/3 on LPL expression by Aβ in SH-SY5Y cells. As shown in Figure 3, with the increase of Aβ concentrations, HDAC2/3 mRNA and protein expression levels were dose-dependently elevated, and there were significantly different between cells exposed to Aβ (2.5–10 μM) and control (*p* < 0.01).

### Dose-Effect Relationships Between Aβ Concentration and miR-29a, PPARγ or LPL Expression Levels as Well as Ace-H3K9 Levels in Their Promoter Region in SH-SY5Y Cells

**Figure 4:**
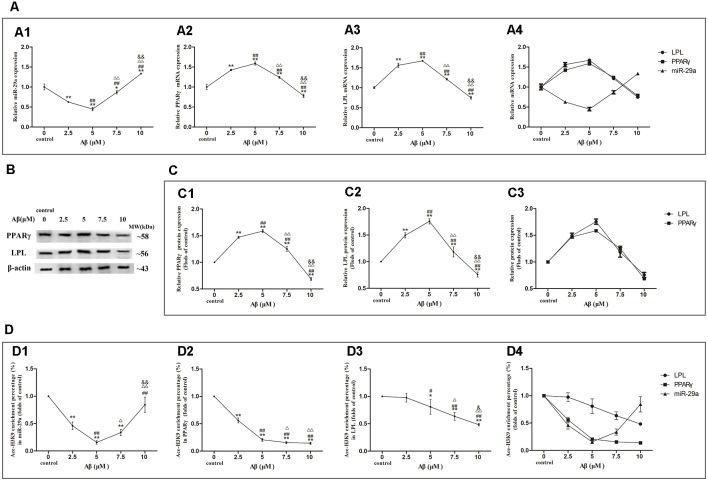
Dose-effect relationships between Aβ concentration and miR-29a, PPARγ, or LPL expression as well as Ace-H3K9 levels in their promoter regions. The relative expression of miR-29a **(A1)** was analyzed by qRT-PCR. U6 was used as a reference standard to normalize microRNA expression. The expression of and PPARγ and LPL mRNA **(A2,A3)** and protein **(B,C1,C2)** were analyzed by qRT-PCR and Western blot, respectively. Panels **(A1–A3)** were integrated into **(A4)**, **(C1,C2)** were integrated into **(C3)**. ChIP-PCR assay was used to measure Ace-H3K9 levels in the promoter regions of miR-29a **(D1)**, PPARγ **(D2)**, and LPL **(D3)** in cells. Panels **(D1–D3)** were integrated into **(D4)** [*n* = 6; mean ± SD; One-way ANOVA followed by LSD multiple comparison tests; *p < 0.05, **p < 0.01 vs. control (0 μm Aβ); ^#^*p* < 0.05, ^##^*p* < 0.01 vs. 2.5 μm Aβ; ^Δ^*p* < 0.05, ^ΔΔ^*p* < 0.01 vs. 5 μm Aβ; ^&^*p* < 0.05, ^&&^*p* < 0.01 vs. 7.5 μm Aβ].

We further detected the expression levels of miR-29a, PPARγ, and LPL as well asAce-H3K9 levels in their promoter region in SH-SY5Y cells exposed to 0, 2.5, 5, 7.5 or 10 μM Aβ (Figures 4D1–D4).

With increasing concentrations of Aβ, the expression levels of miR-29a were decreased first and then increased, and the valley value was reached at 5 μM Aβ (Figures 4A1–A4). Compared with control, miR-29a expression levels were significantly decreased in cells exposed to Aβ (2.5–7.5 μM) and increased in cells with 10 μM Aβ exposure (*p* < 0.01). Moreover, with the increase of Aβ concentrations, LPL and PPARγ expression levels were increased first and then decreased, and the peak value was reached at 5 μM Aβ (Figures 4A–C1–C4). The expression levels of LPL and PPARγ were significantly increased in cells exposed to Aβ (2.5–7.5 μM) and markedly decreased in cells exposed to 10 μM Aβ (*p* < 0.01) compared with control.

As shown in Figures 4D1–D4, with increasing concentrations of Aβ, the levels of Ace-H3K9 in the promoter region of miR-29a were decreased first and then increased, and the valley value was reached at 5 μM Aβ. Compared with control, the levels of Ace-H3K9 in the promoter region of miR-29a were decreased in cells exposed to Aβ (2.5–10 μM; *p* < 0.01). With the increase of Aβ concentrations, the levels of Ace-H3K9 in the promoter region of LPL were dose-dependently decreased, and significant differences were observed between cells exposed to Aβ (5–10 μM) and control (*p* < 0.01). Furthermore, as the concentration of Aβ increased, the levels of Ace-H3K9 in the promoter region of PPARγ were gradually decreased, and then remained stable at 7.5 μM Aβ, and there were significantly different between cells exposed to Aβ (2.5–10 μM) and control (*p* < 0.01).

## Discussion

As a key enzyme in lipid metabolism, LPL plays a role in the hydrolysis of the triacylglycerol component of chylomicrons and very-low-density lipoprotein. Also, several nonenzymatic functions of LPL have recently been identified (Mead et al., 2002; Nishitsuji et al., 2011; Tang et al., 2018). Currently, the results of literature reports on LPL expression level in AD brain are inconsistent (Blain et al., 2006; Wang and Eckel, 2012; Gong et al., 2013; Zhang et al., 2018), which probably due to the reason that LPL expression increases first and then decreases with the progress of AD. It has been documented that LPL in the cell surface binds to Aβ and enhances the cellular uptake of Aβ in a sulfated glycosaminoglycan-dependent manner, and the internalized Aβ is degraded in a lysosomal pathway (Nishitsuji et al., 2011; Tang et al., 2018). Presently, whether intracellular LPL is involved in the degradation of Aβ in the lysosomal pathway is still unknown. Based on the above, we speculate that in the early stage of AD, a small amount of Aβ aggregation may result in a compensatory increase in the expression level of LPL, and thus treated as a molecular chaperone to promote uptake of Aβ for subsequent degradation in the lysosome. With the ever-increasing accumulation of Aβ in AD brain, the expression level of LPL decreases probably due to decompensation, leading to the reduction of Aβ degradation in a lysosomal pathway.

Our previous studies have indicated that the opposite regulation effects of HDAC2 and HDAC3 on LPL expression may play a crucial role in Aβ-induced change of LPL expression (Zhang et al., 2018). This study further confirmed that the level of Ace-H3K9 in the promoter region of LPL was elevated in HDAC3-silenced cells, but remained unaltered in HDAC2-silenced cells. It is suggested that HDAC3 inhibits LPL expression by reducing histone acetylation levels in its promoter region. Furthermore, miR-29a (down-regulated by HDAC2 and up-regulated by HDAC3) mediates the opposite regulation effects of HDAC2 and HDAC3 on LPL expression (Zhang et al., 2018). In the present study, we further found that the level of Ace-H3K9 in miR-29a promoter region was increased in HDAC2-silenced cells, but remained unchanged in HDAC3-silenced cells. These indicate that HDAC2 inhibits miR-29a expression through modulating histone acetylation level in the promoter region of miR-29a, subsequently resulting in an up-regulation of LPL expression, while HDAC3 inhibits LPL expression not only through regulating histone acetylation level in the promoter region of LPL but also by up-regulating miR-29a expression. The regulatory mechanism of HDAC3 on miR-29a expression still needs to be further investigated.

LPL is a downstream target gene of lipid-activated transcription factor PPARγ, which has been reported to be implicated in the progression of AD (Medrano-Jiménez et al., 2019; Chamberlain et al., 2020; Kotha et al., 2020; Xie et al., 2020). This study demonstrated that the expression of PPARγ was decreased in SHSY5Y cells exposed to 2 μM Aβ, and increased in cells with 10 μM Aβ exposure. The above results reported here are consistent with our previous findings that the changes of LPL expression in SH-SY5Y cells exposed to 2 and 10 μM Aβ, respectively (Zhang et al., 2018). It is indicated that PPARγ may have a crucial regulatory effect on the alteration of LPL expression induced by Aβ. Further study is needed to determine the level of PPARγ binding to PPAR response elements in the LPL gene by ChIP assay. This study also confirmed that the expression of PPARγ was reduced in HDAC2-silenced SH-SY5Y cells, but elevated in HDAC3-silenced cells. Also, the level of Ace-H3K9 in the PPARγ promoter region was elevated in HDAC3-silenced SH-SY5Y cells, but remained unaltered in HDAC2-silenced cells. These findings suggest that HDAC2 and HDAC3 are implicated in the effect of Aβ on PPARγ expression, subsequently resulting in the change of LPL expression. Moreover, this study found that the expression of PPARγ was decreased in SH-SY5Y cells treated with miR-29a mimic, and increased in cells treated with miR-29a inhibitor, indicating that miR-29a may also indirectly regulate the transcription of LPL *via* PPARγ/LPL pathway. In the future, it is necessary to confirm whether miR-29a regulates the post-transcription of PPARγ expression by inducing mRNA decay or inhibiting translation. Combining the above results with our previous findings that the expression of miR-29a is increased in HDAC2-silenced SH-SY5Y cells and decreased in HDAC3-silenced cells, it is indicated that miR-29a is involved in the regulation of HDAC2/3 on PPARγ expression.

**Figure 5:**
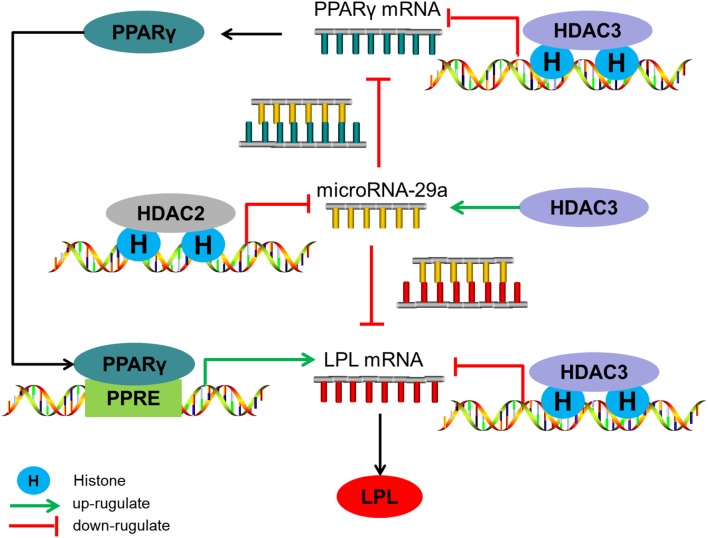
The regulatory mechanism of HDAC2/3 on LPL expression in SH-SY5Y cells. HDAC2 down-regulates microRNA-29a expression by decreasing the level of histone acetylation in its promoter region, subsequently increasing LPL expression directly or through the PPARγ-LPL pathway. HDAC3 decreases LPL expression through inhibiting histone acetylation levels in the promoter region of LPL and PPARγ and up-regulating miR-29a expression which the level of histone acetylation in its promoter region is unaltered.

From what has been discussed above, the level of histone acetylation in the promoter region of miR-29a plays an important role in HDAC2-mediated regulation of Aβ on LPL expression, while HDAC3 mediates the regulation of Aβ on LPL expression through controlling histone acetylation levels in the promoter region of LPL and PPARγ and influencing miR-29a expression (the level of histone acetylation in miR-29a promoter region do not change; summarized in Figure 5).

In the present study, with increasing concentrations of Aβ (0, 2.5, 5, 7.5, 10 μM), the expression of HDAC2/3 was gradually elevated in SH-SY5Y cells, while LPL or PPARγ expression was increased first and then decreased (the dose-effect curve presented an inverse U-shaped trend). Additionally, the level of Ace-H3K9 in the promoter region of miR-29a was down-regulated first and then up-regulated with the increase of Aβ concentrations (the dose-effect curve presented a U-shaped trend), corresponding to the change of miR-29a expression induced by different concentrations of Aβ. These findings suggest that the effects of different concentrations of Aβ on miR-29a expression are mainly through influencing histone acetylation level in the promoter region of miR-29a. As mentioned above, silencing HDAC2 up-regulates miR-29a expression by increasing the level of histone acetylation in the miR-29a promoter region, eventually leading to a decrease of LPL expression (through miR-29a/LPL or miR-29a/PPARγ/LPL pathway). It is indicated that the regulation of HDAC2 on miR-29a may play an important role in up-regulating LPL expression in SH-SY5Y cells exposed to low-dose Aβ. As the concentration of Aβ increased, the expression of HDAC2 was gradually up-regulated, however, the level of histone acetylation in the promoter region of miR-29a was not decreased accordingly. The above findings to some extent support the decreased expression of LPL by high-dose Aβ, while the detailed mechanism of action remains to be further studied. The results from this study showed that Ace-H3K9 levels in the promoter region of LPL and PPARγ were decreased with increasing concentrations of Aβ. Combined with the findings that HDAC3 can reduce histone acetylation levels in the promoter region of LPL and PPARγ, the following indications were obtained: with the increase of Aβ concentration, HDAC3 expression is gradually increased, and then leads to the decrease of histone acetylation levels in the promoter region of LPL and PPARγ, which may be one of the reasons for explaining the down-regulation of LPL expression in SH-SY5Y cells exposed to high-dose Aβ; additionally, HDAC3 can up-regulate the expression of miR-29a, which probably plays a role in decreasing the expression of LPL in cells exposed to high-dose Aβ. It is thus clear that the reason why LPL expression increased first and then decreased may be at least partly the combined effect of HDAC2 and HDAC3.

Here, we speculated that when Aβ is at a lower concentration, it is dominant that HDAC2 inhibits the expression of miR-29a by reducing the level of histone acetylation in miR-29a promoter region, and then up-regulates LPL expression (through miR-29a/LPL or miR-29a/PPARγ/LPL pathway). As the concentration of Aβ increases to a certain level, the level of histone acetylation in the miR-29a promoter region and miR-29a expression was gradually rebound. Combining the above alteration with the down-regulation actions of HDAC3 on histone acetylation levels in PPARγ and LPL promoter region, LPL expression begins to decline. The U-shaped trend of histone acetylation levels in the promoter region of miR-29a plays a leading role in the change of LPL expression with the gradually increasing concentrations of Aβ.

In summary, based on the previous study that the opposite regulation of HDAC2 and HDAC3 on LPL expression, we further revealed the molecular mechanism for LPL expression variations in SH-SY5Y cells exposed to different doses of Aβ. MiR-29a and PPARγ participate in the above regulation, especially the U-shaped change of histone acetylation levels in the miR-29a promoter region plays a crucial role. However, considering the data that we obtained from an *AD* *in* *vitro* neuronal cell models, it should be prudent to extrapolate the conclusions from AD models to humans. In conclusion, this study provides a scientific basis for explaining the molecular mechanism on the alteration of LPL expression in AD.

## Data Availability Statement

The datasets generated for this study are available on request to the corresponding author.

## Author Contributions

LA conceived and designed the experiments. JZ performed the experiments. YL analyzed the data. SW and RQ contributed reagents and materials. JZ, WZ, and LA drafted the manuscript. All authors approved the final version to be published.

## Conflict of Interest

The authors declare that the research was conducted in the absence of any commercial or financial relationships that could be construed as a potential conflict of interest.
